# O.6.1-11 Children’s play and physical activity levels in a natural playground: an observational case study from the Netherlands

**DOI:** 10.1093/eurpub/ckad133.270

**Published:** 2023-09-11

**Authors:** Sander Bliekendaal, Dinand Ekkel

**Affiliations:** Aeres University of Applied Sciences, The Netherlands; s.bliekendaal@aeres.nl; Aeres University of Applied Sciences, The Netherlands; s.bliekendaal@aeres.nl

## Abstract

**Purpose:**

Natural playgrounds stimulate active play in children and, as such, support their healthy development. Therefore, the municipality of Almere (The Netherlands), aims to develop more natural playgrounds within its neighbourhoods and parks. However, insight into how children play in these playgrounds is lacking, which is needed for optimal playgrounds design and policy development. Therefore, this study aimed to map the usage of a typical natural playground in Almere.

**Methods:**

In this observational case study, direct observations (4 days, 4 times a day) of visitors (number, gender, age category, physical activity level, activity) were conducted using the SOPARC method at the natural playground in the Cascade park in Almere (approx. 218.000 inhabitants). This playground (size: 1.5ha) is located at the centre of a neighbourhood and has a natural surface (i.e. grass, sand) and various amenities (e.g. swings, zip-line, slides, climber, hills, sand play area, tiny forest). Descriptive analysis was used to summarize the data. A chi-square test was used to analyze the difference in physical activity levels (sedentary, moderate to vigorous physical activity) between boys and girls.

**Results:**

A total of 287 people were observed at the playgrounds, with an average of 18 people (SD = 15, range: 3-50) per measurement. Most visitors were children (56%), followed by adults (29%), teenagers (14%), and seniors (1%). More girls (56%) were observed and more than half of the children (58%) were playing actively. Physical activity levels of boys and girls were similar (p=.81). The children’s main activities were walking (33%), standing (15%), running (14%), sitting (11%), climbing or sliding (9%), and chasing games (7%).

**Conclusions:**

The playground was mainly used by children, slightly favouring girls. More than half of the children were involved in physically active play, which mostly was a locomotor type of activity. Boys and girls had similar physical activity levels.

**Funding Source:**

This study was supported by a SIA fund (HBOPD.2018.05.57).

